# The SPHERE model: Social Prescribing and Health Equity through Responsive Evaluation

**DOI:** 10.3389/fpubh.2025.1696615

**Published:** 2026-02-18

**Authors:** Bo Kim

**Affiliations:** 1 Center for Health Optimization and Implementation Research, VA Boston Healthcare System, Boston, MA, United States; 2 Department of Psychiatry, Harvard Medical School, Boston, MA, United States

**Keywords:** social prescribing, health equity, policy implementation, social determinants of health, public health evaluation

## Abstract

Social prescribing has emerged as a promising approach to improve health and advance health equity by connecting individuals to non-clinical services that address social determinants of health, such as housing, food access, legal aid, and community-based supports. While referral pathways and link worker roles have received significant attention, far less focus has been given to the public policies that govern access to these services. The success and sustainability of social prescribing efforts depend on whether these enabling policies are well-implemented, community-responsive, and rigorously evaluated, yet few models exist to guide this work. The SPHERE model—Social Prescribing and Health Equity through Responsive Evaluation—addresses this gap. Drawing from implementation science, evaluation theory, and health equity-centered design, SPHERE extends existing implementation frameworks by focusing on how public policies themselves function as implementation infrastructure for social prescribing. The model was refined through considering policy examples and aligning with operational realities of cross-sector, referral-based health systems. SPHERE consists of eight interrelated components: (i) Policy Foundation; (ii) Implementation Infrastructure; (iii) Community Engagement and Co-Production; (iv) Contextual Assessment; (v) Implementation Strategies; (vi) Process Measures; (vii) Outcome Measures; and (viii) Feedback Loops and Learning Mechanisms. These components are grounded in health equity principles and designed to support adaptive and accountable policy delivery. This article illustrates the model’s relevance through three examples of policies that enable social prescribing: Housing First initiatives, Medicaid reentry provisions, and local food policy councils. SPHERE positions policy as a critical, actionable driver of social prescribing systems, helping ensure enabling policies are not only enacted but implemented in community-responsive ways. By integrating SPHERE into the design, implementation, and evaluation of policies that enable social prescribing, governments and communities can transform referral systems into adaptive, health equity-driven platforms for structural change.

## 1 Introduction

Social prescribing has gained traction globally as a strategy to improve health and well-being by connecting individuals with non-clinical supports that address the social determinants of health (SDOH), including access to housing, food, transportation, legal services, and community connection (1, 2). While much attention has focused on developing referral pathways, integrating link workers, and building clinical-community partnerships (3, 4), less attention has been given to the public policies that govern the existence and accessibility of these social supports (5, 6). Without robust policy infrastructure, even the most thoughtfully designed social prescribing systems risk falling short, referring individuals to services that are inaccessible, inconsistently implemented, or unsustainably funded.

Public policies that target SDOH, such as Housing First initiatives, Medicaid reentry provisions, and food access reforms, form the backbone of effective social prescribing (7, 8). These policies determine what resources are available to refer to, under what conditions, and for whom. However, such policies are often unevenly implemented, inadequately evaluated, and insufficiently grounded in health equity considerations (6). This can undermine the reach, credibility, and impact of social prescribing, particularly among under-resourced populations (9).

To address this gap, the SPHERE model—Social Prescribing and Health Equity through Responsive Evaluation—offers a structured, health equity-centered model for guiding the implementation and evaluation of public health policies that support and sustain social prescribing. Grounded in implementation science, evaluation theory, and health equity practice (10–12), the model emphasizes community co-leadership, contextual responsiveness, and continuous learning. It is designed to help policymakers, practitioners, and researchers ensure that policies not only exist, but function as accountable, adaptive systems that truly enable access to non-clinical supports. By focusing on the policy conditions that make social prescribing possible, SPHERE broadens the scope of implementation work from the clinic to the system, advancing a vision of social prescribing not just as a referral infrastructure, but as a pathway to structural change in public health (5, 10).

## 2 Rationale for a structured model

As social prescribing systems expand and mature, their effectiveness increasingly depends on more than just clinical workflows or referral linkages (2, 3). The availability and sustainability of the non-clinical services to which people are referred, such as housing, food assistance, reentry support, and legal aid, are shaped by public policies (9). These policies determine what services exist, how they are funded and governed, and who is eligible to receive them. Without deliberate implementation of such policies, social prescribing programs may falter, offering referrals to resources that are inaccessible or inconsistently delivered (4).

Despite their critical role, these enabling policies are rarely supported by structured models for implementation and evaluation (13). Implementation science offers robust tools for translating evidence into practice, yet most existing models are geared toward programmatic or clinical interventions rather than structural policy change (10). Evaluation science provides methodologies for assessing outcomes, but often emphasizes summative results over the implementation processes that influence health equity and sustainability (5). Health equity-centered design, while offering powerful participatory principles, is not consistently integrated into policy roll-out efforts (12). As a result, public health-enhancing policies intended to support social prescribing often go under-implemented, under-evaluated, and underperforming, particularly for under-resourced populations (6, 14).

SPHERE draws on implementation science, evaluation theory, and health equity practice to provide a structured approach for designing, delivering, and continuously improving the policies that make social prescribing actionable and impactful (11, 15, 16). It is intended to guide various stakeholders—policy developers, health system leaders, community organizations, and researchers—in translating policy commitments into systems that work in practice and work for health equity. SPHERE reframes policy implementation as a core enabler of social prescribing and offers a roadmap for ensuring that the systems supporting SDOH referrals are responsive and built to last (11, 16). Recent national frameworks in Canada, Australia, and the United Kingdom highlight policy-level investment in social prescribing infrastructures (17–19), underscoring global recognition that enabling policies are essential to sustain link worker programs and community referral pathways. SPHERE complements these efforts by offering a structured model for implementing and evaluating such policies.

## 3 Overview of the SPHERE model

SPHERE was developed to provide a structured, health equity-centered model for guiding the implementation and evaluation of public policies that enable effective and accountable social prescribing systems. Recognizing that referral pathways are only as strong as the systems they connect people to, SPHERE shifts attention to the policy conditions that determine whether social prescribing can achieve its goals.

**Figure 1:**
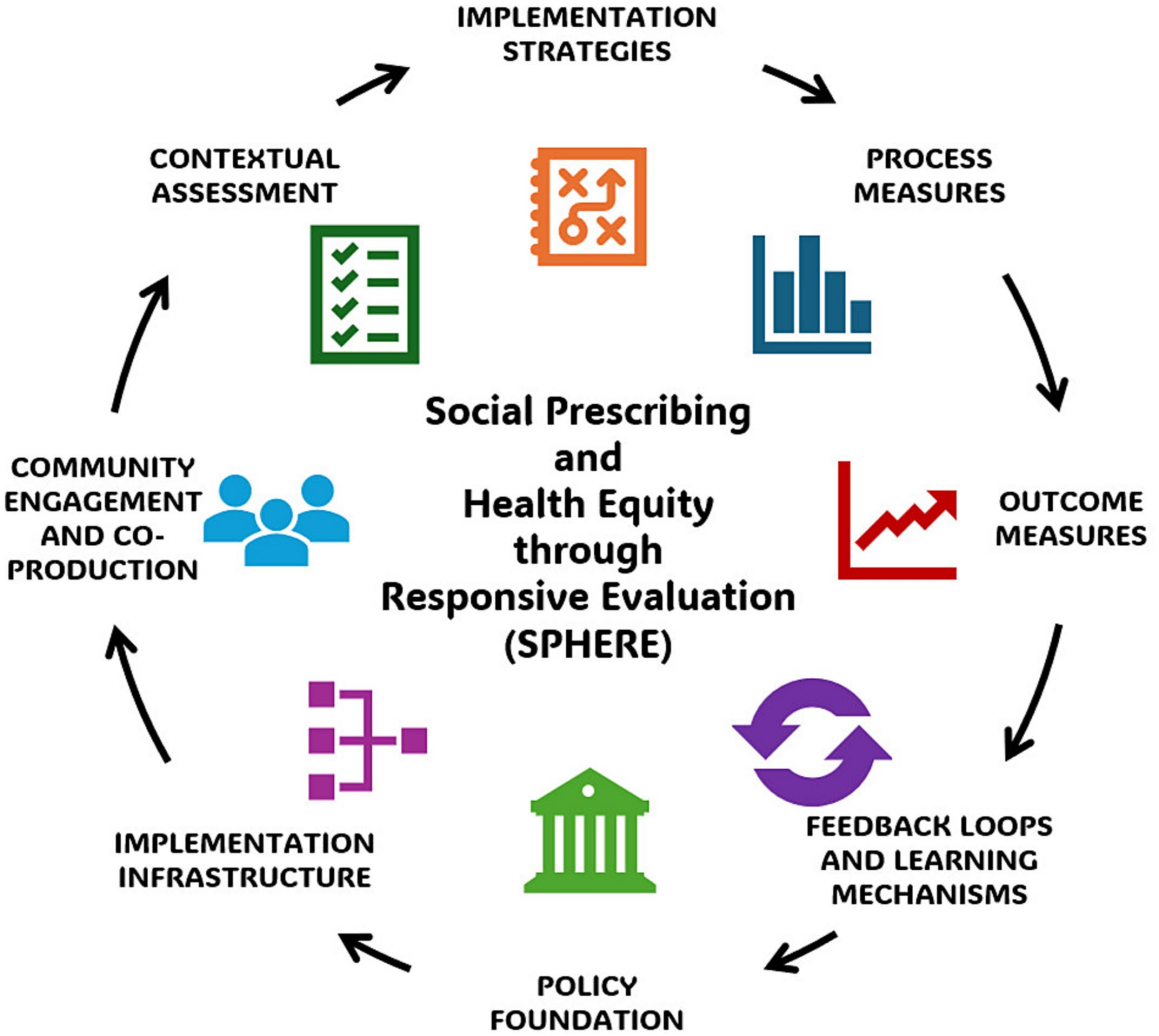
The Social Prescribing and Health Equity through Responsive Evaluation (SPHERE) model. Examples for each model component are: (i) *Policy Foundation*—e.g., state legislation, city ordinances; (ii) *Implementation Infrastructure*—e.g., cross-sector task forces, governance structures; (iii) *Community Engagement and Co-Production*—e.g., community involvement in program design, community-led advocacy; (iv) C*ontextual Assessment*—e.g., policy variations, cultural preferences; (v) *Implementation Strategies*—e.g., staff training, enhanced incentives; (vi) *Process Measures*—e.g., program reach, time to services received; (vii) *Outcome Measures*—e.g., emergency department use, chronic disease risk; (viii) *Feedback Loops and Learning Mechanisms*—e.g., regular feedback forums, iterative policy updates. Together they form a cyclical, adaptive system for health equity-centered social prescribing policy implementation.

Figure 1 provides a visual schematic of SPHERE. The model consists of eight interrelated components, organized to support the real-world implementation and continuous improvement of SDOH-targeting policies that underlie social prescribing. These components emphasize both technical infrastructure and relational practices, and are anchored in principles of health equity, responsiveness, and collaboration.

- *Policy Foundation:* defines the enabling policy or set of policies that aim to address social determinants of health (e.g., housing, food access, Medicaid reentry), and articulates the theory of change by which the policy supports social prescribing outcomes
- *Implementation Infrastructure:* outlines the cross-sector systems, staffing, funding mechanisms, interagency partnerships, and data capacities required to activate the policy in a way that supports referral-based care coordination
- *Community Engagement and Co-Production:* establishes authentic, ongoing approaches for involving community members, especially those with lived experience, in shaping implementation, defining success, and guiding adaptations
- *Contextual Assessment:* calls for proactive assessment of legal, political, organizational, and cultural factors that influence policy uptake and relevance across settings and populations
- *Implementation Strategies:* promotes the selection and tailoring of evidence-informed strategies (e.g., facilitation, training, workflow integration) to increase feasibility, acceptability, and fidelity
- *Process Measures:* supports tracking of core implementation indicators, such as reach, adaptations, and co-production fidelity, to monitor whether and how the policy is functioning as intended
- *Outcome Measures:* encourages measurement of short-, intermediate-, and long-term outcomes linked to policy goals and social prescribing impacts, including disaggregated data to assess effects
- *Feedback Loops and Learning Mechanisms:* establishes structures for real-time learning, adaptive decision-making, and community-informed adjustments that promote sustainability and responsiveness

Together, these components provide a flexible yet structured approach to ensuring that policies are implemented in ways that strengthen, rather than strain, social prescribing systems. SPHERE is not meant to be prescriptive; rather, it is intended to support dynamic and collaborative implementation efforts across a range of policy domains and delivery contexts.

SPHERE can be applied as a practical, five-step guide to strengthen the policy environment surrounding social prescribing:

1. Define the enabling policy that supports social prescribing referrals (e.g., housing, nutrition, justice reentry) and articulate its theory of change
2. Identify cross-sector infrastructure (e.g., funding streams, workforce roles, data systems) needed for policy activation
3. Co-design implementation strategies with community partners and link worker teams
4. Track process and outcome measures, including health equity-disaggregated indicators of referral reach and service uptake
5. Establish feedback loops for continuous learning between policymakers, practitioners, and communities

Development of companion decision aids and self-assessment checklists is planned to assist social prescribing systems in applying each step.

## 4 Application of the SPHERE model: illustrative use cases

Table 1 outlines three illustrative examples that demonstrate how SPHERE can be used to strengthen the implementation of public policies that support social prescribing. Further description of each example follows the table. The three illustrative cases were purposefully selected to reflect distinct social prescribing domains—housing, justice, and food access—each linked to a policy that functions as referral infrastructure. This variation demonstrates how SPHERE’s eight components can guide the implementation and evaluation of policies that sustain social prescribing pathways across levels of governance.

**Table 1:** Illustrative examples demonstrating how the SPHERE model—Social Prescribing and Health Equity through Responsive Evaluation—can be used to strengthen the implementation of public policies that support social prescribing.

Housing First policy as an enabler of social prescribing for homelessness	Medicaid reentry policy as a platform for justice-focused social prescribing	Local food policy councils supporting food security prescriptions
Social prescribing initiatives that serve people experiencing homelessness often include referrals to housing services; a Housing First policy, which prioritizes access to permanent supportive housing without preconditions, establishes the infrastructure that makes these referrals possible and meaningful	Justice-involved individuals are increasingly included in social prescribing programs; Medicaid reentry policies that allow benefits to be reactivated before release create a vital bridge to medical care, behavioral health, and other services upon reentry	Social prescribing programs that include food insecurity screening often refer individuals to nutrition support services; local food policy councils shape the regulatory, economic, and spatial conditions that determine whether healthy, affordable food is available where it is needed most
**SPHERE Component 1: Policy Foundation**		
Municipal or state-level adoption of Housing First as a standard	Federal waiver or state legislation authorizing Medicaid coverage during reentry planning	City ordinances or resolutions establishing a food policy council
**SPHERE Component 2: Implementation Infrastructure**		
Cross-sector housing-health task forces; coordinated entry systems	Embedded benefits navigators; corrections-Medicaid data-sharing agreements	Governance structures linking agriculture, health departments, and community groups
**SPHERE Component 3: Community Engagement and Co-Production**		
Peer support specialists and tenants involved in program design	Input from formerly incarcerated individuals and community health workers	Resident-led food planning initiatives and youth-led advocacy
**SPHERE Component 4: Contextual Assessment**		
Local barriers such as housing stock shortages and landlord discrimination	Variation in jail policies, provider capacity, and stigma-related barriers	Geographic food access mapping; cultural dietary preferences; retail zoning rules
**SPHERE Component 5: Implementation Strategies**		
Training for housing providers; incentives for landlords; shared case management tools	Staff training; automated enrollment systems; continuity-of-care planning	Support for mobile markets; urban agriculture policy changes; healthy corner store initiatives
**SPHERE Component 6: Process Measures**		
Time to housing placement; fidelity to Housing First principles; referral closure rates	Enrollment completion pre-release; linkage to primary or behavioral health care within 30 days	Implementation of council recommendations; program reach by neighborhood
**SPHERE Component 7: Outcome Measures**		
Housing stability; emergency department use; mental health outcomes	Service engagement; medication adherence; recidivism rates	Improved food access; reduced nutrition-related chronic disease risk; increased community ownership
**SPHERE Component 8: Feedback Loops and Learning Mechanisms**		
Regular tenant feedback forums; real-time dashboard monitoring; interagency coordination meetings	Joint review meetings between corrections, Medicaid, and community partners to inform improvements	Quarterly public forums; participatory evaluations; iterative policy agenda updates

### 4.1 Housing First policy as an enabler of social prescribing for homelessness

The Housing First policy exemplifies how SPHERE can guide the structured implementation of policies that enable social prescribing (20, 21). As many social prescribing programs aim to connect individuals experiencing homelessness with housing supports, the actual availability and accessibility of these supports are directly shaped by local Housing First policies (20). SPHERE can help operationalize this connection by prompting policymakers and practitioners to not only adopt such policies but also build the implementation infrastructure required, such as coordinated entry systems and housing-health integration platforms (22). Through SPHERE’s emphasis on community engagement, people with lived experience of homelessness can be involved in shaping service delivery and ensuring relevance (20). The model also highlights the importance of contextual assessment (e.g., housing market shortages or local zoning constraints) and can guide the selection of implementation strategies like landlord incentives and peer navigation (22). Process and outcome measures, such as housing placement time, service uptake, and tenant retention, can be tracked and disaggregated to identify challenges and course-correct through real-time feedback (21). Thus, SPHERE can transform Housing First from policy intent to an accountable, health equity-driven foundation for housing-related social prescriptions.

### 4.2 Medicaid reentry policy as a platform for justice-focused social prescribing

SPHERE can provide critical guidance for implementing Medicaid reentry policies in ways that maximize their role as structural enablers of social prescribing for justice-involved populations. While social prescribing pathways increasingly seek to address the health and social needs of individuals leaving incarceration, successful referrals to medical care, substance use treatment, and housing services depend on Medicaid coverage being reactivated in a timely and coordinated manner (23, 24). SPHERE can ensure that this policy function is fully realized by guiding investment in implementation infrastructure, such as data-sharing agreements, embedded benefits navigators, and coordination between corrections and Medicaid agencies (24, 25). Community co-production can be integrated through including formerly incarcerated individuals in planning and decision-making, while contextual assessment can address local variation in jail systems, stigma, and reentry resources (25). SPHERE can support the use of tailored strategies, such as pre-release enrollment workflows and continuity-of-care planning, and can call for evaluation measures that track not just enrollment rates but also outcomes like care continuity and recidivism (23, 24). With feedback loops built into policy oversight, SPHERE can help ensure that Medicaid reentry provisions meaningfully support social prescribing’s goal of sustained reentry support (25).

### 4.3 Local food policy councils supporting food security prescriptions

Local food policy councils serve as a compelling example of how SPHERE can be applied to ensure that food-related social prescriptions are supported by a responsive policy ecosystem (26). Social prescribing programs that address food insecurity often refer individuals to community food resources, but whether those resources are sufficient and geographically accessible depends on the local food policy environment. SPHERE emphasizes that this environment must be actively shaped through structured policy design and collaborative governance (26). In the case of food policy councils, SPHERE can guide the development of implementation infrastructure, such as cross-sector coalitions and sustainable funding for nutrition initiatives, and can mandate meaningful community engagement, including resident-led food planning and youth participation (26). Through contextual assessment, councils can identify transportation barriers, retail gaps, and regulatory hurdles that limit food access (27). SPHERE-aligned strategies, such as zoning reforms to enable urban agriculture or support for mobile markets, can then be deployed to expand food access (27, 28). The model also ensures that process and outcome measures, like reach of food programs, affordability, and nutrition-related health improvements, are tracked with a health equity lens and used to inform adaptive governance. By aligning food policy governance with social prescribing needs, SPHERE can enable structural solutions to food insecurity that are accountable to the communities they are meant to serve.

These illustrative examples show how SPHERE can be applied flexibly across policy areas to ensure that public health policies are implemented in ways that reinforce and extend the reach of social prescribing. Comparable national experiences in Canada, Australia, and the United Kingdom demonstrate that social prescribing initiatives succeed when supported by coherent, health equity-focused policy frameworks (17–19). Common facilitators include cross-sector coalitions linking health, housing, and social services; community co-production; and shared outcome monitoring. Persistent barriers, such as fragmented governance and uneven resource distribution, underscore the need for structured policy implementation models like SPHERE that integrate infrastructure, measurement, and learning systems into social prescribing design. By embedding health equity, contextual fit, and community leadership into each aspect, the model supports policies that do more than authorize services—they build trust, dismantle barriers, and enable structural change.

## 5 Discussion

SPHERE offers a timely and actionable model for addressing a growing implementation gap in the social prescribing movement: how to ensure that public policies are effectively deployed and continuously improved to support non-clinical referral systems. As health systems increasingly integrate social prescribing into care pathways, the importance of strong and responsive policy foundations becomes more evident and more urgent.

### 5.1 Implications for public health policy, practice, and research

For policymakers, SPHERE reframes policy not as a static directive, but as a dynamic system that must be supported with thoughtful infrastructure, collaborative governance, and embedded accountability (29). It highlights the need to move beyond policy enactment to implementation planning, resource alignment, and health equity-informed performance monitoring (29, 30). The model helps policymakers anticipate barriers, mobilize partnerships, and design feedback procedures that support learning and responsiveness (31).

Importantly, SPHERE encourages policy design that explicitly ensures that policies enabling social prescribing do not bypass or underserve those who need them most (32). Recent analyses of national social prescribing programs (17–19) reveal that even well-intentioned enabling policies can generate uneven impacts when implementation lacks accountability or community input. SPHERE’s embedded feedback loops and participatory evaluation elements are designed to identify and correct these variations in real time, ensuring that policy translation advances rather than undermines health equity.

In particular, within social prescribing systems, health equity functions at three levels: as a principle shaping goals and resource allocation; as a process operationalized through participatory design, inclusive referral networks, and disaggregated monitoring; and as an outcome reflected in measurable reductions in uneven access and health outcomes. SPHERE integrates all three dimensions to ensure that social prescribing policies produce health equity-enhancing structural change.

Public health and social prescribing practitioners often find themselves referring individuals to community resources that are overstretched or inconsistently applied. SPHERE can help practitioners identify when these challenges stem not from referral workflows, but from gaps or misalignments in policy (33). By applying the model, practitioners can participate in policy feedback loops, engage in cross-sector coalitions, and advocate for reforms that make social prescribing more sustainable (29, 33). It also offers a structure for aligning internal quality improvement with external systems change (34).

For researchers and evaluators, SPHERE provides a conceptual model for studying the intersection of policy, implementation, and health equity. It invites the use of mixed-methods approaches to assess not only whether enabling policies improve health, but how they are adopted, for whom they work, and under what conditions they succeed (35). The model supports health equity-centered implementation science by calling for disaggregated data, participatory methods, and attention to adaptation and power dynamics (30, 35). Operationally, this means that SPHERE treats health equity not only as a guiding value but as a measurable process and outcome that is tracked through the existence of specific disaggregated indicators, participatory governance structures, and accountability procedures, respectively. Researchers can use SPHERE to evaluate policy uptake in real-world social prescribing systems and generate evidence that is both theoretically grounded and practically useful (31).

In sum, SPHERE offers a structure for ensuring that the policies supporting social prescribing systems are not only well-designed, but also meaningfully implemented and evaluated. By connecting policy change to health, the model reinforces social prescribing as a scalable strategy for structural transformation in public health.

### 5.2 Limitations and areas for further development

While SPHERE offers a comprehensive and flexible model for guiding the implementation of policies that support social prescribing, it remains conceptual and subject to several important limitations. Notably, a key risk is overgeneralization across vastly different policy contexts; SPHERE should be viewed as a heuristic to be adapted rather than a one-size-fits-all model. The specific limitations listed below further highlight the need for continued development, empirical validation, and contextual adaptation.

First, SPHERE has not yet been applied prospectively in implementation or evaluation studies. While its structure is grounded in theory and informed by practice-based illustrations, its real-world utility remains to be tested across various policy domains and delivery contexts. Future work should prospectively apply SPHERE within active social prescribing systems—such as community link worker networks, primary care-based referral pathways, or municipal well-being hubs—to examine how the model supports implementation, measurement, and health equity outcomes. Mixed-methods evaluations will be essential for determining feasibility, adaptability, and added value for social prescribing policy and practice (36).

Second, many public health and community-based organizations involved in social prescribing may lack the capacity or resources to engage in full-scale policy implementation planning and responsive evaluation. While SPHERE is designed to be adaptable, practical guidance is needed to support tiered or modular use, especially in low-resource environments (37). This includes tools to help communities prioritize model components, tailor strategies to local conditions, and scale up efforts incrementally. In other words, low-resource environments may require simplified or modular adaptations of SPHERE components; identifying feasible minimal-core elements is an important future direction.

Third, SPHERE is designed to complement existing social prescribing infrastructure, not replace it. However, additional work is needed to clarify how the model aligns with referral platforms, workforce models (e.g., link workers or community health workers), and clinical integration strategies (38). Toolkits that help implementers bridge SPHERE with operational frameworks already in use could enhance usability and uptake.

Fourth, the success of SPHERE depends on political will, cross-sector collaboration, and institutional readiness to share power and invest in health equity. In settings where these conditions are weak or contested, some of the model’s more transformational elements, such as community co-governance or feedback-driven policy redesign, may be difficult to realize (37, 39). Structural barriers commonly observed in social prescribing policy efforts, such as short political cycles, fragmented interagency mandates, and constrained analytic capacity, can limit adoption of models like SPHERE. These challenges highlight the importance of tailoring implementation plans to local readiness while maintaining a commitment to co-governance and health equity principles.

Addressing these limitations presents opportunities for empirical pilot-testing of SPHERE through mixed-methods evaluations and learning collaboratives that document feasibility, adaptation, and outcomes across various policy settings (40). As the field of social prescribing matures, models like SPHERE will need to evolve in tandem with practice—remaining grounded in values, but responsive to the realities of structural change (36).

### 5.3 Conclusion

As social prescribing gains prominence as a strategy to improve health by addressing social determinants, attention must turn to the policies that enable or constrain its impact. The promise of social prescribing depends not only on effective referral pathways or dedicated link workers, but on the existence of strong and well-implemented public policies that ensure access to the non-clinical services individuals are being referred to. Without intentional strategies to implement and evaluate these enabling policies, social prescribing systems risk being built on unstable foundations.

SPHERE offers a conceptual roadmap for strengthening the policy and governance foundations of social prescribing. By integrating health equity, contextual assessment, and continuous learning into the implementation of enabling policies, the model identifies leverage points for future empirical work. Ongoing testing within live social prescribing systems will determine how effectively SPHERE translates conceptual guidance into sustained community outcomes. Ultimately, SPHERE invites a broader vision of social prescribing—not simply as a tool for connecting patients to community resources, but as a lever for advancing structural change in public health.

SPHERE advances beyond existing implementation frameworks by positioning policy implementation itself as a core necessity for strengthening social prescribing systems. By connecting the dots between policy design, referral infrastructure, and adaptive evaluation, SPHERE provides actionable guidance for policymakers and practitioners. The next phase involves empirical validation in partnership with health systems and communities actively delivering social prescribing, to refine the model and measure its contribution to enhanced health and well-being.

## Data Availability

The original contributions presented in the study are included in the article, further inquiries can be directed to the corresponding author.
